# The Organisation of the Profession in Ireland

**Published:** 1914-02-14

**Authors:** 


### THE PROFESSION OF MEDICINE.

### The Organisation of the Profession in Ireland.

THE membership of the British Medical Association in Ireland has never comprised the same proportion of the practitioners there as it has in Great Britain; and it is freely stated that the recent raising of the subscription on the part of the Association will result in many resignations of Irish members. There is, however, another professional organisation in Ireland: that is the Irish Medical Association, which up to the present date has published its own periodical, the *Journal of the Irish Medical Association.* This Association also undertakes, besides the supervision of the general interests of the medical profession, the functions which in Britain are discharged by the Medical Defence Union and similar bodies. It employs a secretary and pays its councillors' travelling expenses; and with all these outgoings has contrived to make ends meet on an annual subscription of one guinea.

The *Journal* is published monthly, and contains little beyond reports of the Association's meetings: scientific contributions have not been accepted, though a proposal to include them has been mooted from time to time. The production and distribution of the *Journal* costs just under six shillings per member per annum at present. But to enlarge it so as to include scientific papers and to publish it weekly would no doubt very greatly increase this sum. Accordingly, the Council of the Association has decided to cease the publication of the *Journal;* and in its place to distribute to its members the *Medical Press and Circular,* a paper which has always paid especial attention to Irish medical affairs. The proprietors of the *Medical Press* have agreed to distribute it to every member for half a guinea a year, a minimum membership of 450 being guaranteed. They have also agreed to issue an Irish Supplement fortnightly, this to be edited at the expense of the Irish Medical Association.

This proposal was accepted by the Council after an animated discussion, at which much difference of opinion was expressed. It appeared that the proprietors of the *Lancet* would have arranged to supply the members of the Irish Medical Association with that paper, and also with an Irish Supplement weekly for sixteen shillings a year per member. This exceedingly advantageous offer commended itself more to some of those present than the terms offered by the *Medical Press,* but was not acceptable to the majority.

There can be little doubt that the Council is wise in abandoning its own *Journal* and coming to terms with some paper of standing and influence. It is not for us to comment upon the relative advantages of the offers submitted to the Council by British medical newspapers, but it is of some interest to note that fears are expressed lest the medical defence work of the Association should languish for lack of funds through the increased expenditure now sanctioned by the Council. It is also noteworthy that one of the reasons given for the change of policy is the chance of capturing for the Irish Medical Association some of those who are (stated to be) contemplating retirement from the British Medical Association. Ireland possesses such a different version of National Insurance from that which applies to Britain that the difficulties of the medical profession there are probably very different in many ways from those which beset British practitioners. But we may be sure that difficulties there will be, and a plentiful crop of them; so it is highly important that the organisation of the profession should be as thorough, and its courage as resolute, as they ought to be over here. In any case, the editor of the *Medical Press* can be trusted to do his best to make that paper representative of all that is most worthy in the Irish medical world; and we shall watch the career of our contemporary in its new *rôle* with a sympathetic interest.

### ABUSES OF PANEL PRACTICE.

At a recent meeting of the Manchester Insurance Committee one of the members narrated the results of a tour of inspection which he had carried out one evening during the very cold weather. At seven o'clock in the evening, he found surgeries stuffed with people, many of them spitting and coughing, and others standing outside. The doctors were turning them out as quickly as possible; and it was impossible for each individual to get proper attention.

There is, of course, nothing in this which is extraordinary or unusual; the same sort of thing is happening all over the kingdom. But the important point is that Insurance Committee-men are beginning to realise what panel practice means. When this knowledge reaches the mass of the members of these Committees possibly something may be done to remedy the injustice to the insured of the panel system. That, however, has not yet happened, though it can only be a matter of time before it does.

In these columns instances have frequently been given of the real inwardness of surgery practice under the Insurance Act; so the revelations from Manchester are no surprise to us. Indeed, we can without difficulty quote much worse examples of negligent, inefficient, and downright cruel behaviour on the part of panel practitioners. Only the other day a gentleman who holds an important position of trust vouched to us for the truth of an incident which seems almost incredible. His servant fell ill, and decided to visit the panel doctor, for the first time. After waiting in a crowded surgery for some time, the patient obtained an audience of the doctor; and then discovered that to economise time the latter had two patients in his consulting-room together. The dismay of anyone thus confronted with the necessity of confiding full details of an illness, not only to the doctor, but also to another patient within earshot, can be more readily imagined than described. We do not hesitate to describe the doctor's conduct as outrageous and indecent. Presumably he found that no patient stayed very long in such humiliating surroundings, and that his "surgery hour" was rapidly got through. It would not be fair to regard this abominable conduct as typical of panel practice or panel doctors; but at least it could not occur except under the Insurance Act and its offspring, the panel system.

### DEFICIENCY OF SANATORIA.

The sanatorium benefit provisions of the Insurance Act came into force, it will be remembered, six months before those regarding medical benefit in general. They have now been established, therefore, more than eighteen months; and, after all allowances are made for the delays inevitable in getting so complicated a scheme into working order, things certainly are most disappointingly backward in many districts. The *Daily Express* has been devoting much attention to the deficiencies in the sanatorium treatment as provided under the Act, and some perfectly horrible stories of hardship and even death have come to light, due to the failure of certain Insurance Committees to carry out their duty to the insured.

One such case is that of a Kensington young man, the chief breadwinner of a family consisting of his father and mother and four or five young brothers and sisters. In October this young man suddenly developed consumption, and was attended, after some delay, by a panel doctor. After many weeks of waiting for sanatorium benefit, he was eventually directed to present himself for examination, and was then given to understand that he would shortly be admitted to a sanatorium. For weeks more he waited, growing steadily worse in health, not even getting the extra nourishment which Mr. Lloyd George used to make so much of; and it was only when a local clergyman warned the Insurance Committee of the risk they were running by procrastination that he received an order for admission to the Victoria Park Hospital, in the East End of London. Here he afterwards died.

This case is scandalous enough; but it would obviously be unfair to draw general inferences from a single incident, however disgraceful. Unfortunately, it does not stand alone. In Gloucestershire, for example, there are (outside Gloucester and Bristol) some 720,000 persons in the Insurance Committee's area. Of these about 175,000 are insured, 1,200 of whom are tuberculous. For the treatment of these patients some 35 beds are all that are available so far; and stories just as painful as that quoted above from Kensington are coming to light from Gloucestershire.

